# Removal of Portions of Three Dorsal Vertebræ for Paralysis from Fracture

**Published:** 1830-01-01

**Authors:** 


### XXIX.

### REMOVAL OF PORTIONS OF THREE DORSAL VERTEBRÆ FOR PARALYSIS FROM FRACTURE.

The following case is recorded in the number of our valuable Transatlantic Cotemporary, the North American Surgical Journal, for July of the present year. It is published in the form of an extract from a letter of the operator Alban G. Smith, M.D. of Danville, to Dr. B. H. Coates.

"About two years since, I was called a distance of about twelve miles, to a place called Pleasant Hill, the residence of a community of the people commonly called Shakers, to see a young gentleman, a member of the society, who had fallen from a horse, and had become paralysed in all the extremities with the exception of the muscles above the elbow joint. Upon examining the spinous processess of the vertebræ, I found that one of them was thrown to the right side, to the distance of about a quarter of an inch. Concluding that the whole of the vertebra, body and all, was dislocated, I told him that, in all probability, the case would soon terminate fatally; and merely directed some cups to be applied to the parts adjacent, with low diet, and some gentle cathartics. Since then I heard but little of the case, except that he was still alive, until, a few weeks ago, Dr. Thomas, the physician of the society, and his brother came to see me, at the earnest request of the patient, stating his importunities, that if I thought there was a possible chance of relief by any process, I would attempt it; as death was far preferable to his present situation. They informed me, at the same time, that Dr. Dudley had been called to see the case, had made an incision down to the bone, and had given it as his opinion, that the body of the bone was not dislocated, but that one side of the base of the spinous process was broken and pressed in on the spinal mrarow. Having implicit confidence in an opinion emanating from such a justly distinguished surgeon, I told them that I would come down, and if upon examination of the case, the state of his general health appeared to admit of any probability of its success, I would attempt to remove the pressure. Accordingly, in a few days, I went down; and after I had represented to him the few chances of success, the difficulties of removing a part so tied down without fatal injury to the spinal marrow, and also the probability of the injury sustained by the spinal marrow itself being so extensive that the powers of nature might not be sufficient to resuscitate it, he replied that, if there was one chance in a thousand of its being of any kind of advantage to him, he wanted the attempt to be made. After sending for my friend, the accurate anatomist, Dr. H. Miller, who concurred with me as to the propriety of the operation, I proceeded. I made an incision, about five or six inches in length, along the spinal ridge, and then two deep incisions, transversely to, and at each extremity of the former, and three inches and a quarter in length, all down to the bone. I then dissected all the muscles and ligaments as far as the heads of the transverse processes, and scraped the bone clean. The situation of the parts was found as Dr. Dudley had described them; the fragments being pressed to one side, but so completely united, and so smooth on the upper surface that the line of separation was not very well marked. I next took a small Hey's saw, and made an incision on both sides, as near the base of the transverse processes as I could, as deep as I could with safety to the spinal marrow and the nerves going off at the sides, and long enough to cut through the bone above, about half way, which was the third dorsal vertebra. I then sawed off the end of the transverse process of the second, and into the transverse process of the third, about half its base. Then, taking a very strong tooth forceps, with its ends turned on one side, and getting a claw in each incision made by the saw, I was enabled to break up the external plate, and part of the internal; so that I could now, with a small strong pair of pliers or forceps, break, by a small piece at a time, the whole of the bone that I had cut round with the saw. Here the anatomist will see the difficulties with which I had to contend; as the parts are so firmly and securely tied down with ligaments. Thus it will be seen that I took out a part of the spinous processes of two vertebræ, half of the third, and the whole of the fourth; the fourth being the bone which seemed to be the most depressed. I now brought the parts together, putting a tent at the bottom; and put him to bed. He had a chill, succeeded by a fever, and some bilious symptoms, which were carried off by a dose of calomel.

"I saw him in a week afterwards. The ulcers in his gluteal muscles, which were produced by lying on his back, were healing up; and he had some additional sensation in his hands. Since then I have not seen him; but I entertain considerable hopes.

"I wrote the above several weeks ago: I have since received two letters from his attending physician, and have sent my student down; by all which means I am informed that sensation is returning as rapidly as he can bear it. The pain of returning feeling is so great that he complains very much. It is something like the sensation experienced when 'one's leg is asleep,' as it is vulgarly expressed. His general health is good; and I have but little doubt of his being soon entirely restored to the use of his muscles. Should I ever be called to a case of the kind again, I would advise an immediate operation."

Since the date of that report, "feeling has got down into the patient's thighs," much to his own delight and the surgeon's gratification. The case is of so interesting a nature that we thought it right to transfer it to our pages, although we candidly confess that we differ from the enthusiastic author in the conclusions to which he has come. We believe that there are very few cases indeed of fractured spine which justify an operation, and not one in a thousand that would be bettered by its performance. In the instances of recent injury who can tell that the arch only is broken or depressed, the medulla or its membranes unscathed, and the body of the bone unbroken? We know, by dissections, that the body most frequently *is* implicated, and who, we ask, aware of this fact, would venture on a tedious, doubtful, and coarse operation? Cæteris paribus WE would not. In the present case, however, this step was had recourse to for the *secondary* rather than the primary consequences of the spinal injury, and so far it would appear with a certain degree of success. In the nearly analogous injuries of the head the secondary symptoms have sometimes required and been benefited by the use of the trephine, but they have not seldom been aggravated or the patient even destroyed by its employment. Of this we witnessed an instance last year. If the operation then on the head under such circumstances maintain an ambiguous character, how much more precarious must be that upon the spine! The true bearing of the case appears to us to be this:—In the majority of the cases of really severe spinal injury the patient will not survive the primary stage; but if he should do so the presumption is that he will either recover from the effects of the mischief, or remain more or less paralytic for a time, and ultimately sink exhausted, whilst in neither contingence will an operation be proper. There may be one or two rare combinations of circumstances, such perhaps as in the present instance, vindicating the employment of the trephine or saw, but we believe that these are Oases in the desert, and that the great, the very great proportion of cases are not of this pleasing description.

